# Chemokine CXCL14 is associated with prognosis in patients with colorectal carcinoma after curative resection

**DOI:** 10.1186/1479-5876-11-6

**Published:** 2013-01-07

**Authors:** Jun Zeng, Xudan Yang, Lin Cheng, Rui Liu, Yunlong Lei, Dandan Dong, Fanghua Li, Quek Choon Lau, Longfei Deng, Edouard C Nice, Ke Xie, Canhua Huang

**Affiliations:** 1 The State Key Laboratory of Biotherapy, West China Hospital, Sichuan University, Chengdu, 610041, P. R. China; 2 Sichuan Academy of Medical Science, Sichuan Provincial People’s Hospital, Chengdu, 610041, P. R. China; 3 School of Life Sciences and Chemical Technology, Ngee Ann Polytechnic, 535 Clementi Road, Clementi, Republic of Singapore; 4 Monash University, Department of Biochemistry and Molecular Biology, Clayton, Victoria, 3800, Australia; 5 Department of Oncology, Sichuan Academy of Medical Sciences, Sichuan Provincial People’s Hospital, Chengdu, 610041, People’s Republic of China

**Keywords:** Colorectal carcinoma, CXCL14, Disease-free survival, Overall survival, Prognosis

## Abstract

**Background:**

The chemokine CXCL14 has been reported to play an important role in the progression of many malignancies such as breast cancer and papillary thyroid carcinoma, but the role of CXCL14 in colorectal carcinoma (CRC) remains to be established. The purpose of this study was to investigate the expression pattern and significance of CXCL14 in CRC progression.

**Method:**

265 colorectal carcinoma specimens and 129 matched adjacent normal colorectal mucosa specimens were collected. Expression of CXCL14 in clinical samples was examined by immunostaining. The effect of CXCL14 on colorectal carcinoma cell proliferation was measured by MTT assay, BrdU incorporation assay and colony formation assay. The impact of CXCL14 on migration and invasion of colorectal carcinoma cells was determined by transwell assay and Matrigel invasion assay, respectively.

**Results:**

CXCL14 expression was significantly up-regulated in tumor tissues compared with adjacent nontumorous mucosa tissues (*P* < 0.001). Tumoral CXCL14 expression levels were significantly correlated with TNM (Tumor-node-metastasis) stage, histodifferentiation, and tumor size. In multivariate Cox regression analysis, high CXCL14 expression in tumor specimens (n = 91) from stage I/II patients was associated with increased risk for disease recurrence (risk ratio, 2.92; 95% CI, 1.15-7.40; *P* = 0.024). Elevated CXCL14 expression in tumor specimens (n = 135) from stage III/IV patients correlated with worse overall survival (risk ratio, 3.087; 95% CI, 1.866-5.107; *P* < 0.001). Functional studies demonstrated that enforced expression of CXCL14 in SW620 colorectal carcinoma cells resulted in more aggressive phenotypes. In contrast, knockdown of CXCL14 expression could mitigate the proliferative, migratory and invasive potential of HCT116 colorectal carcinoma cells.

**Conclusion:**

Taken together, CXCL14 might be a potential novel prognostic factor to predict the disease recurrence and overall survival and could be a potential target of postoperative adjuvant therapy in CRC patients.

## Background

Colorectal carcinoma (CRC) is one of the most common malignancies worldwide [1]. Over 1 million new cases of colorectal carcinoma are diagnosed each year [2]. In China, colorectal carcinoma ranks fifth among cancer deaths, with the incidence increasing annually [3]. Despite improved radiotherapeutic and chemotherapeutic regimens and surgical outcomes, almost half of the colorectal carcinoma patients relapse within 5 years of treatment and inevitably succumb to the disease. The prognosis and treatment of colorectal carcinoma currently depends on the pathologic stage of disease at the time of diagnosis and primary surgical treatment. Unfortunately, disease stage alone does not allow accurate prediction of outcome for individual patients [4]. If patient outcomes are predicted more accurately, treatments could be tailored to avoid undertreating patients destined to relapse or overtreating patients who could be cured by surgery alone. Thus, new biomarkers pivotal to tumor biology are urgently needed to improve prognosis of the adjuvant treatment regimens.

Chemokines, a family of small (8–14 KDa) secreted proteins that orchestrate leukocyte trafficking, have been implicated in tumor development and progression [5-7]. They exert their cellular effect by specifically activating corresponding transmembrane G protein-coupled receptors [8]. Chemokines are classified into the C, CC, CXC, and CX_3_C families, based on variations in a structural motif of conserved N-terminal cysteine residues. Approximately 50 chemokines and 20 chemokine receptors have been identified. Chemokines could promote the invasiveness of cancer cells by triggering integrin clustering and enhancing their adherence to extracellular matrix via their receptors [9,10]. Furthermore, chemokines and chemokine receptors could also serve as prognostic factors for cancer outcomes [11,12].

CXCL14 (originally identified as *BRAK*, *BMAC*, or *Mip2γ*) is a chemokine with as yet unknown function. This molecule was first identified by differential display using normal epithelial cells and head and neck squamous carcinoma cells and was shown to be expressed in many other normal tissues and in various tumors of epithelial origin with heterogeneous expression levels [13-16]. As a unique member of the chemokine family, the receptor for CXCL14 has not yet been identified and the role of this molecule in tumor progression is controversial. Although the role of CXCL14 as a tumor suppressor seemed well established, recent studies suggested that CXCL14 might actually promote tumor progression [17,18]. For example, CXCL14 was reported to be up-regulated in pancreatic cancer tissues compared to chronic pancreatitis and normal pancreas [19]. It has also been documented that CXCL14 transcript is markedly higher in papillary thyroid carcinoma than in adjacent noncancerous tissues and positively correlated with lymph node metastasis [20]. CXCL14 also acts as a multi-modal stimulator of prostate and stomach tumor growth [15,21]. These findings necessitate a re-evaluation of the function of CXCL14 in epithelial tumors.

Till now, there has been little data suggesting the association between CXCL14 and colorectal carcinoma. This study was therefore designed to investigate the expression and clinical significance of CXCL14 in colorectal cancer. Our results showed that elevated CXCL14 expression in primary colorectal cancers was associated with poor disease-free survival and overall survival, indicating CXCL14 might be used as a potential prognostic marker for CRC patients.

## Materials and methods

### Cell culture

HCT116, SW620, RKO and LoVo colorectal cancer cell lines were purchased from American Type Culture Collection (ATCC, Rockville, MD). Cells were maintained in Dulbecco’s Modified Eagle’s Medium (DMEM, Invitrogen) containing 10% heat-inactivated fetal calf serum (Hyclone, Logan, UT), penicillin (10^7^ U/L) and streptomycin (10 mg/L) at 37°C in a humidified chamber containing 5% (v/v) CO_2_ in air.

### Cloning of CXCL14, transfection and semi-quantitative RT-PCR

To clone the CXCL14 cDNA, we isolated total RNA from HCT116 cell line. First strand cDNA was reversely transcribed from 1 μg of total RNA in a final volume of 20 μl using reverse transcriptase and random hexamers with ExScript™ reagent kit (TaKaRa, Dalian, China) according to the manufacturer’s instructions. Primers were designed using Primer Premier 5 software. Primers used were CXCL14: forward 5^′^-CGG GAT CCA TGT CCC TGC TCC CAC GCC-3^′^, reverse 5^′^-CCC TCG AGC TAT TCT TCG TAG ACC CTG CGC TT-3^′^ (336 bp product fragment). PCR was performed with rTaq (TaKaRa) in a DNA thermal cycler (Bio-Rad) according to a standard protocol as follows: initial denaturation (2 min at 94°C) followed by 30 cycles of denaturation at 94°C for 30 s, annealing at 56°C for 45 s, and elongation at 72°C for 50 s. After the last cycle a terminal elongation step (5 min at 72°C) was added and thereafter the samples were kept at 4°C. PCR products were run on 2.0% agarose gel, stained by SYBR Gold (Molecular Probes, Eugene, OR), and analyzed under UV light.

To construct a eukaryotic expression vector, the CXCL14 cDNA was cloned into pcDNA3.1(+) plasmid using the Eukaryotic TA Expression kit (Invitrogen Life Technologies, Grand Island, NY) according to the manufacturer’s instructions. Human colorectal cell line SW620 was transfected with the human CXCL14 expression vector using Lipofectamine 2000 (Invitrogen Life Technologies, Grand Island, NY) according to the supplier’s instructions. Next, transfected cells were split and treated with G418 (Invitrogen Life Technologies, Grand Island, NY), and 12 clones were obtained. SW620 cells stably transfected with pcDNA3.1(+) empty vector was used as mock control. Validation of the difference of CXCL14 expression among colorectal cell lines was performed by semi-quantitative RT-PCR. Gene transcription of GAPDH and CXCL14 was analyzed by a two-step reverse transcription-PCR as above.

### Construction of and transfection with shRNA expressing vector targeting CXCL14

A 21-mer shRNA expressing vector targeting CXCL14 (shCXCL14) and its scrambled sequence-expressing vecotr as a negative control (shNC) were synthesized following the published literature [22]. The insert sequence for CXCL14 shRNA was 5^′^-GCA CCA AGC GCT TCA TCA ACT GTG AAG CCA CAG ATG GGT TGA TGA AGC GCT TGG TGC-3^′^ and that for the scramble control shRNA was 5^′^-GCC ATA CGC GAC ATA ACC TCT GTG AAG CCA CAG ATG GGA GGT TAT GTC GCG TAT GGC-3^′^. The underlined nucleotides indicated the 19-bp hairpin loop sequence. HCT116 cells were transfected with those vectors by use of a Lipofectamine. Cells treated with Lipofectamine alone were used as a mock control. For proliferation and motility assays, cells were transfected with those vectors for 24 h before use. For expression analyses, cells were harvested at 48 h post transfection.

### Western blotting

Proteins were extracted in RIPA buffer (50 mM Tris-base, 1.0 mM EDTA, 150 mM NaCl, 0.1% SDS, 1% TritonX-100, 1% Sodium deoxycholate, 1 mM phenylmethylsulfonyl fluoride) and quantified using the DC protein assay kit (Bio-Rad, USA). Samples were separated on 10% or 12% SDS-PAGE and transferred to PVDF membranes (Amersham Biosciences). The membranes were blocked overnight with PBS containing 0.1% Tween 20 in 5% skimmed milk at 4°C and subsequently probed using the following primary antibodies: rabbit-anti-CXCL14 (diluted 1:400, Proteintech). Blots were incubated with the respective primary antibodies for 2 h at room temperature and washed 3 times in TBS with Tween 20. Subsequently, the blots were incubated with secondary antibodies (diluted 1:10,000; Santa Cruz Biotechnology) conjugated to Horseradish Peroxidase for 2 h at room temperature. Target proteins were detected by enhanced chemiluminescence reagents (Amersham Biosciences, Piscataway, NJ), as previously described [23]. β-actin was used as an internal control.

### In vitro proliferation assays

5-Bromo-2^′^-deoxyuridine (BrdU) incorporation assay, was performed as previously described [24]. Cells were seeded in 24-well culture plates at a density of 5 × 10^5^, grown to preconfluence (60%). After treatment, BrdU (10 mM) (Roche Applied Science, Indianapolis, IN) was added to each well and incubated for 4 h. Anti-BrdU antibody (Zhongshan Biothechnology, Zhongshan, China), appropriate fluorescein isothiocyanate-labeled secondary antibody were used. Staining was visualized using confocal microscope (Leica, Heidelber, Germany). Cell proliferation was determined by counting the number of BrdU-positive cells in eight alternative areas. 3-(4,5-methylthiazol-2-yl)-2,5-diphenyl-tetrazolium bromide (MTT) assay was performed as described previously [25]. For evaluation of cell growth in soft agar, cells (3000 cells/well) were resuspended in DMEM containing 10% FBS with 0.35% agarose and layered on top of 0.5% agarose in DMEM on 6-well plates. For colony formation assay, SW620 cells (100 cells/well) were resuspended in DMEM 10% FBS with rhCXCL14 (PeproTech) (0-100 ng/ml) on 6-well plates. Cultures were maintained for 14 days and plates were stained with Crystal Violet. Each experiment was done in triplicate.

### In vitro motility assays

Cell migration assays were performed in 24-well transwell chambers (Corning, NY). Cells in serum-free medium (1 × 10^5^ cells per well) were added to the upper chamber. After 20 h, the number of cells that migrated through the membrane to the lower chamber was counted. For invasion assays, matrigel (1:3, BD) was added to the transwell membrane chambers, incubated for 4 h, and seeded with cells. Cells, which had migrated to the lower chamber, were counted after 72 h, as previously described [26].

### Surgical specimens

265 primary colorectal tumors and 129 corresponding adjacent normal colorectal mucosa of the same subjects, were collected from the patients who underwent surgical resections during 2006 at the Sichuan Provincial People’s Hospital (Chengdu, China). The primary tumors were staged according to tumor-node-metastasis (TNM) classification system of the International Union against Cancer [27]. Tumor differentiation was graded according to Edmondson Steiner grading by experienced pathologists. Further clinicopathologic patient information was extracted from the clinical notes and a summary of the detailed colorectal cancer demographics is listed in Table 1. Informed consent for tissue procurement was obtained from all patients prior to analysis, and the project was approved by the Institutional Ethics Committee of Sichuan University.

**Table 1:** Colorectal cancer demographics

**Characteristics**	**Value**
Total No. of patients*	265
Age, y	
Median	61
Range	25-88
60 y or younger, n	116
Sex, n	
Male	134
Female	131
Surgical specimens*	294
Primary	265
AJCC stages	
294p I/II	106
p III/IV	159
Lymph node metastasis	29
Tumor invasion^#^	
pT1	2
pT2	13
pT3	235
pT4	15
Lymph node metastasis^#^	
pN0	40
pN1	172
pN2	53
Pathologic grade^#^	
Well	68
Moderate	164
Poor	33
Tumor size, cm^#^	
<2	74
2–5	154
>5	37
Location^#^	
Colon	134
Rectum	131

Abbreviation: AJCC, American Joint Committee on Cancer.

*Ninety patients had paired adjacent normal colorectal mucosa specimens.

^#^Primary colorectal cancer tumors.

### Immunohistochemical staining

Tissues were formalin-fixed and paraffin-embedded, and serial 4 μm thickness sections were taken for immunohistochemistry analysis using a Dako Envision System (Dako Cytomation GmbH, Hamburg, Germany Denmark) according to the manufacturer’s instructions. Briefly, the paraffin sections were deparaffinized, rehydrated, incubated in 3% H_2_O_2_ for 10 min in the dark at room temperature to block the endogenous peroxidase activity, and antigen-retrieved in citrate buffer (pH 6.0) using autoclave sterilizer method. Subsequently, the sections were preincubated with normal fetal calf or goat serum diluted in PBS (pH 7.4) for 15 min at 37°C, followed by incubation at 4°C overnight with the primary antibodies (rabbit anti-CXCL14, dilution 1:50, Proteintech; rabbit anti-CXCL14, dilution 1:200, Abcam; rabbit anti-Ki67, dilution 1:200, Abcam). After rinsing in fresh PBS, slides were incubated with horseradish peroxidase-linked goat anti-rabbit antibodies at 37°C for 40 min, followed by staining with 3,3^′^-diaminobenzidine (DAB) substrate solution (Dako Cytomation GmbH) and counterstaining with Mayer’s hematoxylin. Non-immune rabbit IgG at the same dilution as the primary antibody was used as the negative controls.

### Evaluation of immunohistochemical staining

Cells with visible brown particles in the cytoplasm were regarded as positive. All sections were evaluated by two senior pathologists (Y.X. and D.D.), blinded to patient outcomes and all clinicopathologic findings. The immunohistochemical staining was evaluated based on the intensity (weak = 1, intense = 2) of CXCL14 immunostaining and the density (0% = inverse, 1-50% = 1, 51-75% = 2, >76% = 3) of positive tumor cells [28]. The final scores of each sample were multiplied intensity and density, and the tumors were finally determined as inverse: score = 0; low expression: score ≤ 3; high expression: score>3. If the evaluations did not agree the specimens were reevaluated and then classified according to the assessment given most frequently by the observers.

### Statistical analysis

Analysis was performed with SPSS 16.0 for Windows (SPSS Inc); qualitative variables were compared using the Pearson χ^2^ Test and Fisher’s exact test; and quantitative variables were analyzed by the *t* test. A Spearman test for non-parametric variables was used to evaluate correlation between clinical factors and CXCL14 expression. Survival curves for colorectal cancer patients with high or low CXCL14 expression levels were constructed using the Kaplan-Meier analysis. Univariate analysis of clinical factors including age, sex, TNM stage, tumor invasion, lymph node metastases, histodifferentiation, location and tumor size was assessed by the log-rank test. Multivariate analysis of disease-free survival and overall survival were performed by the Cox proportional hazards regression model using the forward stepwise method when clinical prognostic factors were adjusted. The results were considered statistically significant when *P* value was less than 0.05.

## Results

### CXCL14 expression was up-regulated in CRC

**Figure 1:**
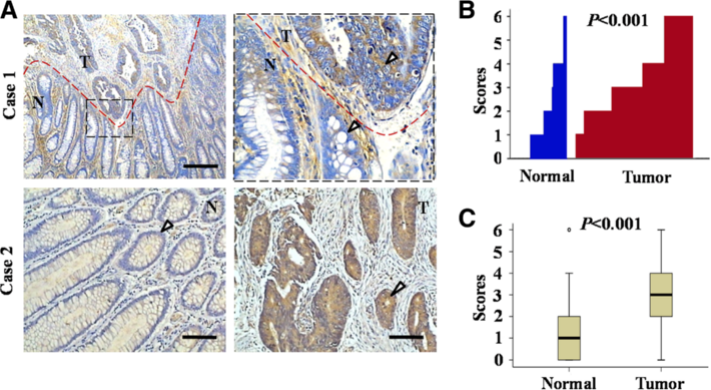
***A, *CXCL14 expression was elevated in human colorectal cancer.** Individual FFPE sections (e.g. case 1) demonstrated normal colorectal mucosa, with adjacent colorectal cancer that had invaded underneath the normal mucosa. *Inset in the left panel*, the junction between normal mucosa and adjacent tumor, shown at a higher magnification in the *right panel*, demonstrated the differences in the level of CXCL14 expression. ‘T’ refers to tumor tissue and ‘N’ refers to adjacent non-tumor tissue from the same patient. Bar, 100 μm. Paired samples of normal colorectal tissue and colorectal tumor tissue from the same patients (e.g. case 2) demonstrated little detectable CXCL14 immunoreactivity in the normal colorectal mucosa. In contrast, the colorectal tumor demonstrated intense CXCL14 immunostaining in the cytoplasm of the tumor. Bar, 50 μm. ***B***, CXCL14 expression was plotted using the immunohistochemical scores in each carcinoma and adjacent normal tissues. ***C***, CXCL14 expression scores were shown as box plots, with the horizontal lines representing the median; the bottom and top of the boxes representing the 25^th^ and 75^th^ percentiles, respectively; and the vertical bars representing the range of data.

To investigate the potential clinical role of chemokine CXCL14 in colorectal cancer, immunohistochemistry staining was performed on 265 colorectal cancer specimens and 129 matched adjacent normal colorectal mucosa specimens with an antibody to CXCL14, the specificity of which we first confirmed (Additional file 1: Figure S1A). Strong CXCL14 staining was mainly observed in the cytoplasm of tumor cells, while partially in the normal mucosa (Figure 1A). CXCL14-positive staining was observed in 54.3% (70/129) of normal colorectal mucosa samples, while in 85.3% (226/265) of primary CRC samples. CXCL14 expression was markedly up-regulated in colorectal tumor tissues compared with normal colorectal mucosa (*P* < 0.001, Figure 1B, C). Further, high CXCL14 immunoreactivity was also found in colorectal cancer tissues, compared to adjacent non-cancerous tissues, by using another antibody recognizing CXCL14, which may rule out the possibility of non-specific staining (Additional file 1: Figure S1B). Most of the stroma cells were CXCL14-negative, although sporadic positive staining on these cells was also observed. These analyses suggested that up-regulation of CXCL14 might be involved in colorectal cancer initiation and progression.

### CXCL14 was correlated with several clinicopathologic factors in CRC

**Figure 2:**
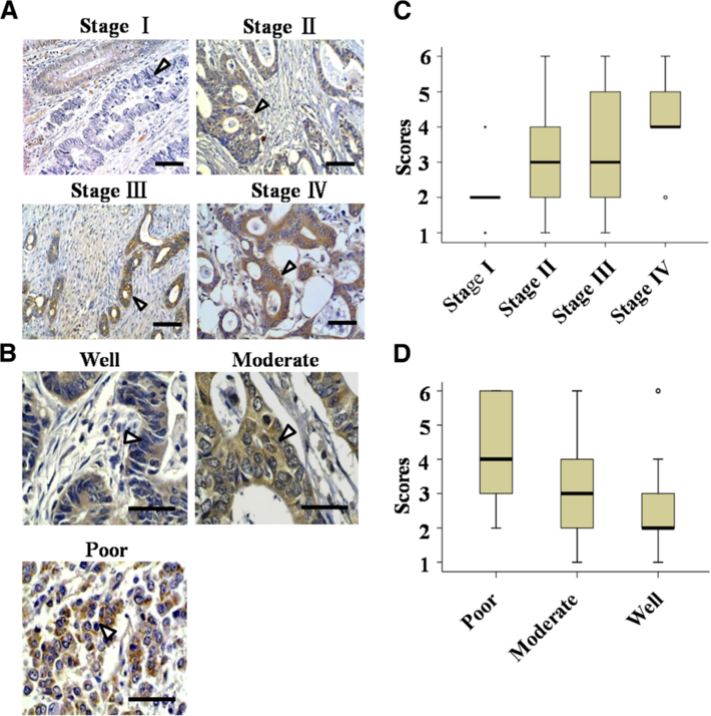
**The level of CXCL14 expression was significantly correlated with TNM stage and histodifferentiation. *A, ***strong immunoreactivity was more likely to present with late-stage CRC compared to early-stage CRC (*P* < 0.001). Bar, 50 μm. ***B, ***poorly differentiated colorectal tumors showed stronger immunoreactivity of CXCL14 than moderately and well differentiated colorectal tumors (*P* = 0.002). Bar, 25 μm. ***C*** and ***D, ***comparison of immunohistochemical scores of CXCL14 according to TNM stage (***C***) and histodifferentiation (***D***).

We next analyzed the relevance between CXCL14 expression in tumor tissues and clinicopathologic factors. The parameters included in this analysis were sex (male, female), age (25–88 y), TNM stage (Stages I, II, III, IV), tumor invasion (T1, T2, T3, T4), histodifferentiation (well-, moderately and poorly differentiated), lymph node metastasis (N0, N1, N2), tumor size (<2, 2–5, >5) and location (colon, rectum). We found that the levels of CXCL14 expression were positively associated with TNM stage (*P* < 0.001, Figure 2A, C; Table 2), and reversely associated with histodifferentiation (*P* = 0.002, Figure 2B, D; Table 2). Of the 226 CXCL14-positive CRC cases, 8 were in Stage I (submucosa invasion), 83 in Stage II (subserosal invasion), 127 in Stage III (lymph node metastasis) and 8 in Stage IV (distant metastasis); and 57 were well-differentiated, 140 moderately and 29 poorly differentiated. In addition, the levels of CXCL14 expression were positively correlated with tumor size (*P* = 0.001, *P* = 0.003 for early-stage (I/II) colorectal carcinoma and late-stage (III/IV) colorectal carcinoma, respectively, Table 3). No apparent correlation between CXCL14 expression and other clinical factors was observed (Table 3). These analyses indicated that up-regulation of CXCL14 in colorectal cancer cells was correlated with tumor progression.

**Table 2:** Relation between clinical factors and CXL14 expression in all colorectal carcinoma patients

**Clinical Factors**	**CXCL14 Expression**		
	**Low**	**High**	**P value**
TNM stage			<0.001^※^
p I	7 (3.1%)	1 (0.4%)	
p II	62 (27.4%)	21 (9.3%)	
p III	66 (29.2%)	61 (27.0%)	
p IV	2 (0.9%)	6 (2.7%)	
Grade			0.002^※^
Well	41 (18.1%)	16 (7.1%)	
Moderate	86 (38.1%)	54 (23.9%)	
Poor	10 (4.4%)	19 (8.4%)	

^※^ χ2 test.

**Table 3:** **Comparison of clinical factors with CXCL14 for stage I**/**II patients and stage III**/**IV patients**

**Clinical Factors**	**CXCL14 Expression (stage I/II)**			**CXCL14 Expression (stage III/IV)**		
	**Low (n = 9)**	**High (n = 22)**	***P *value**	**Low (n = 68)**	**High (n = 67)**	***P *value**
Sex			0.599^※^			0.543^※^
Female	27 (29.7%)	10 (11.0%)		34 (25.2%)	37 (27.4%)	
Male	42 (46.2%)	12 (13.2%)		34 (25.2%)	30 (22.2%)	
Age			0.878^†^			0.878^†^
Mean ± SD	62.4 ± 13.5	63.7 ± 14.1		58.7 ± 14.7	59.5 ± 14.0	
Median	65	64		61	62	
Range	25–88	35–82		25–84	29–88	
TNM stage			0.674^∮^			0.165^∮^
p I	7 (7.7%)	1 (1.1%)				
p II	62 (68.1%)	21 (23.1%)				
p III				66 (48.9%)	61 (45.2%)	
p IV				2 (1.5%)	6 (4.4%)	
Tumor invasion		0.123^∩^				0.522^∩^
pT1	1 (1.1%)	0 (0%)				
pT2	6 (6.6%)	1 (1.1%)		2 (1.5%)	2 (1.5%)	
pT3	59 (64.8%)	18 (19.8%)		64 (47.4%)	61 (45.2%)	
pT4	3 (3.3%)	3 (3.3%)		2 (1.5%)	4 (3.0%)	
Lymph node No./metastasis		0.348^∩^				0.78^∩^
0-7/pN0	20 (22.0%)	5 (5.5%)		2 (1.5%)	6 (4.4%)	
8–12/pN1	37 (40.7%)	12 (13.2%)		56 (41.5%)	49 (36.3%)	
>12/pN2	12 (13.2%)	5 (5.5%)		10 (7.4%)	12 (8.9%)	
Grade			0.246^∩^			0.05^∩^
Well	21 (23.1%)	7 (7.7%)		20 (14.8%)	9 (6.7%)	
Moderate	42 (46.2%)	8 (8.8%)		44 (32.6%)	46 (34.1%)	
Poor	6 (6.6%)	7 (7.7%)		4 (3.0%)	12 (8.9%)	
Tumor size, cm		0.001^∩^				0.002^∩^
<2	20 (22.0%)	0 (0%)		26 (19.3%)	11 (8.1%)	
2-5	45 (49.5%)	18 (19.8)		34 (25.2%)	38 (28.1%)	
>5	4 (4.4%)	4 (4.4%)		8 (5.9%)	18 (13.3%)	
Location			0.100^※^			0.191^※^
Colon	27 (29.7%)	13 (14.3%)		34 (25.2%)	41 (30.4%)	
Rectum	42 (46.2%)	9 (9.9%)		34 (25.2%)	26 (19.3%)	

^∮^ Fisher’s exact test; ^†^ t test; ^∩^Nonparametric correlations; ^※^ χ2 test.

### CXCL14 was correlated with disease recurrence

**Figure 3:**
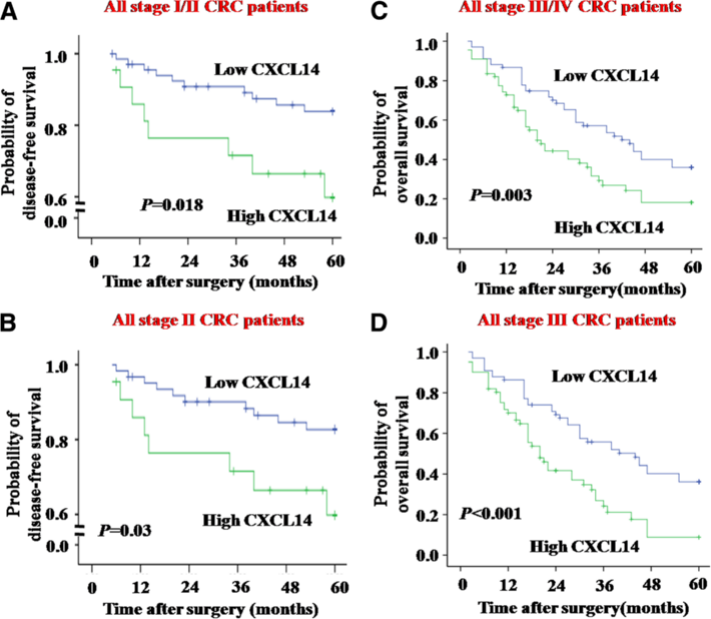
**Cumulative disease**-**free and overall survival of CRC patients with high or low CXCL14 expression. *A*** and ***B, ***Kaplan-Meier disease-free survival curves to estimate survival of AJCC stage I/II CRC patients (*A*) and AJCC stage II CRC patients (*B*). ***C*** and ***D, ***Kaplan-Meier overall survival curves to estimate survival of AJCC stage III/IV CRC patients (*C*) and AJCC stage III CRC patients (*D*). Comparison was made of groups with high CXCL14 and low CXCL14 expression. Marks on graph lines represent censored samples. *P* value refers to two-sided log-rank tests.

Patients with early-stage (I/II) colorectal carcinoma were divided into low-CXCL14-expressing tumor group (n = 69) and high-CXCL14-expressing tumor group (n = 22). Analysis by the Kaplan-Meier method revealed a significant decrease in disease-free survival of high CXCL14-expressing patients compared with low CXCL14-expressing patients: 36.4% *v* 14.5% (Log-rank *P* = 0.018, Figure 3A). By univariate analysis, CXCL14 expression, number of lymph nodes, and tumor size were significantly correlated with disease recurrence (Table 4). To determine whether the prognostic value of CXCL14 expression was independent of other risk factors associated with clinical outcome of colorectal carcinoma, multivariate analysis was performed using Cox proportional hazard model. The results showed only CXCL14 remained a prognostic factor in disease-free survival of early-stage colorectal carcinoma patients (risk ratio, 2.92; 95% CI, 1.15-7.40; *P* = 0.024; Table 4). Among all the AJCC stage II CRC patients, high CXCL14 expression was found in 21 samples while expression of CXCL14 was relatively low in other 62 samples. Those with tumors that highly expressed CXCL14 also showed significantly poorer disease-free survival (Log-rank *P* = 0.03, Figure 3B).

**Table 4:** **Univariate and multivariate analyses of stage I**/**II disease**-**free survival and stage III**/**IV overall survival**

	**Univariate Analysis**		**Multivariate Analysis**		**Univariate Analysis**		**Multivariate Analysis**	
**Clinical Factors**	**Recurrences/No. of Patients (stage I/II)**	**Log**-**Rank *P***	**Risk Ratio (95%CI)**	**Wald *P***	**Deaths/No. of Patients (stage III/IV)**	**Log-Rank *P***	**Risk Ratio (95%CI)**	**Wald *P***
Sex		0.314	1.0	NS		0.337	1.0	NS
Female	9/37				42/71			
Male	9/54				43/64			
Age, years		0.999	1.0	NS		<0.001	3.753	<0.001
<70	13/65				58/99		(1.746-8.070)	
≥70	5/26				27/36			
TNM stage		0.222	1.0	NS		0.084	1.0	NS
p I	0/8							
p II	18/83							
p III					82/127			
p IV					3/8			
Tumor invasion		0.408	1.0	NS		0.842	1.0	NS
pT1	0/1							
pT2	0/7				3/4			
pT3	18/77				79/125			
pT4	0/6				3/6			
Lymph node NO. /metastasis		0.006	1.0	NS		<.001	4.450	<.001
0-7/pN0	9/25				2/8		(2.675-7.401)	
8–12/pN1	4/48				65/105			
>12/pN2	5/18				18/22			
Grade		0.329	1.0	NS		0.132	1.0	NS
Well	6/28				18/29			
Moderate	8/50				55/90			
Poor	4/13				12/16			
Tumor size, cm		0.049	1.0	0.099		0.249	1.0	NS
<2	2/20				25/37			
2-5	12/63				42/72			
>5	4/8				18/26			
Location	0.164	1.0	NS			0.440	1.0	NS
Colon	11/40				44/75			
Rectum	7/51				41/60			
CXCL14		0.018	2.92 (1.15-7.40)	0.024		0.003	3.087 (1.866-5.107)	<0.001
Low	10/69				39/68			
High	8/22				46/67			

Abbreviation: NS, not significant.

### CXCL14 was correlated with overall survival

The level of CXCL14 expression in primary colorectal carcinoma specimens from 135 late-stage (III/IV) colorectal carcinoma patients stratified these tumors as 67 high- and 68 low-CXCL14-expressing tumors. An overall survival analysis of 135 colorectal cancer patients indicated that the level of CXCL14 expression was inversely correlated with survival time of the patients after surgery. Patients with strong CXCL14 expression showed significantly decreased overall survival. The overall survival curves were constructed by the Kaplan-Meier method and analyzed using Log-rank test (Log-rank *P* = 0.003, Figure 3C). To determine the prognostic significance of CXCL14 as a predictor of overall survival in patients with late-stage colorectal carcinoma, univariate and multivariate analyses were conducted. By univariate analysis, clinical factors including age, lymph node metastasis and CXCL14 expression were significantly correlated with overall survival (Table 4). Factors that were significant in univariate analysis and showed a trend toward significance were included in the multivariate model. Results showed CXCL14 expression could be used as a prognostic factor in overall survival of colorectal carcinoma patients (risk ratio, 3.087; 95% CI, 1.866-5.107; *P* < 0.001; Table 4). Patients with stage III CRC were divided into low-CXCL14-expressing tumor group (n = 66) and high-CXCL14-expressing tumor group (n = 61). By univariate analysis, CXCL14 expression (low expression v high expression) remained significantly different for all stage III CRC patients (Log-rank *P* < 0.001, Figure 3D).

### CXCL14 up-regulation was correlated with high proliferation in CRC

**Figure 4:**
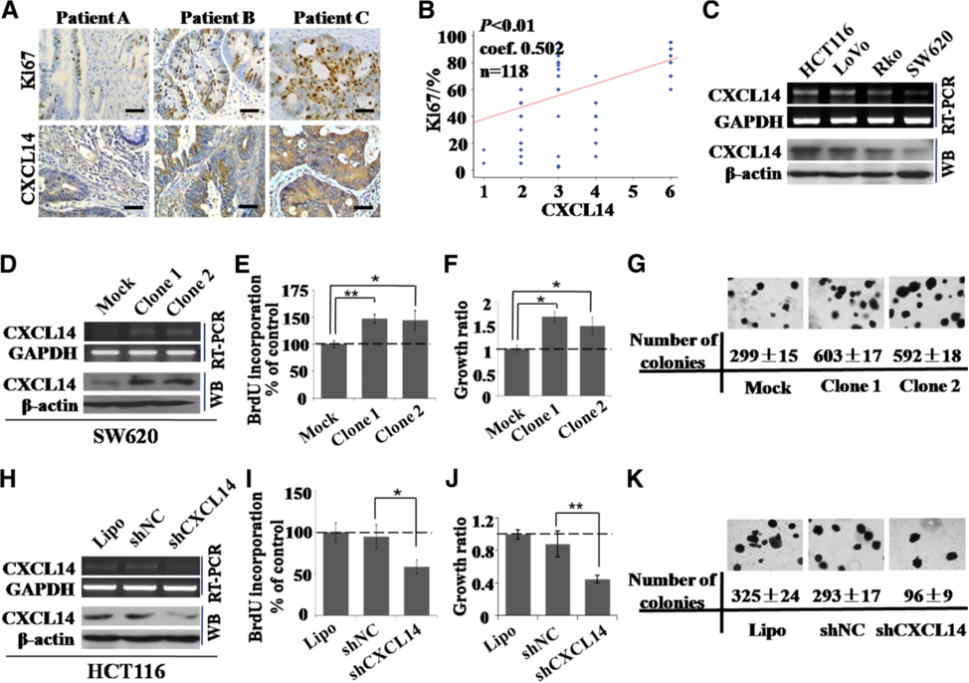
**Effects of CXCL14 expression on human colorectal cancer cell proliferation. *A, ***immunohistochemical staining of human colorectal tumor sections using CXCL14 and Ki67 antibodies. Bar, 25 μm. ***B, ***correlation between the expression levels of CXCL14 and Ki67 (n = 118; *P* < 0.01, two-tailed Spearman test coefficient: 0.502). ***C, ***endogenous CXCL14 mRNA and protein levels were determined by semi-quantitative RT-PCR and Western blotting in several colorectal cancer cell lines. ***D, ***CXCL14 was stably over-expressed in the SW620 subclones (clone 1 and clone 2). ***E***-***G, ***proliferation of SW620 cells stably expressing CXCL14 or mock vector was compared by BrdU incorporation assay (*E*), MTT assay (*F*), and colony formation assay (*G*), respectively. ***H, ***expression of CXCL14 in HCT116 cells was inhibited by transfection with shCXCL14. ***I***-***K, ***proliferation of HCT116 cells transfected with shCXCL14 or shNC was measured by BrdU assay (*I*), MTT assay (*J*), and colony formation assay (*K*), respectively. Error bars represent ± SD. Student’s *t* test was used for the statistical analyses. *, P < 0.05; **, P < 0.01.

To explore whether CXCL14 is correlated with the proliferation of colorectal cancer cells, levels of Ki67, a marker of cell proliferation [29], were analyzed in 118 resected colorectal cancer specimens, which were also examined with an antibody to CXCL14 (Figure 4A). CXCL14 and Ki67 levels were plotted against each other, and statistical analysis using the Spearman’s correlation test showed significant correlation between these parameters (*P* < 0.01; Figure 4B). These results suggested that CXCL14 might play a causal role in the induction of proliferation in colorectal cancer.

### Effect of CXCL14 on colorectal cancer cell proliferation in vitro

To explore the effect of CXCL14 on colorectal cancer cell proliferation, we investigated the expression levels of CXCL14 mRNA and protein in four different colorectal cancer cell lines: HCT116, SW620, RKO and LoVo (Figure 4C). The results showed that SW620 had only low levels of endogenous CXCL14 expression and thus human CXCL14 cDNA was transfected into the cell line, with G418 to allow the formation of colonies. Two clones (clone 1 and clone 2) with elevated CXCL14 were selected for further studies. Semi-quantitative RT-PCR and Western blotting confirmed that CXCL14 was stably over-expressed compared with mock control (Figure 4D). MTT assay and BrdU incorporation assay indicated that the two subclones grew significantly faster than control cells (Figure 4E, F). We performed a colony formation assay in 6-well plates. As expected, the results showed that the two subclones yielded increased colonies compared with control cells (Figure 4G). In addition, treatment with rhCXCL14 protein could promote SW620 cell proliferation (Additional file 2: Figure S2A-C). While shRNA-mediated inhibition of CXCL14 expression in HCT116 cells caused a significant decrease in cell proliferation (Figure 4H-K). Collectively, these data identified previously unrecognized growth-stimulatory effects of CXCL14 on colorectal cancer cells.

### Effect of CXCL14 on colorectal cancer cell motility in vitro

**Figure 5:**
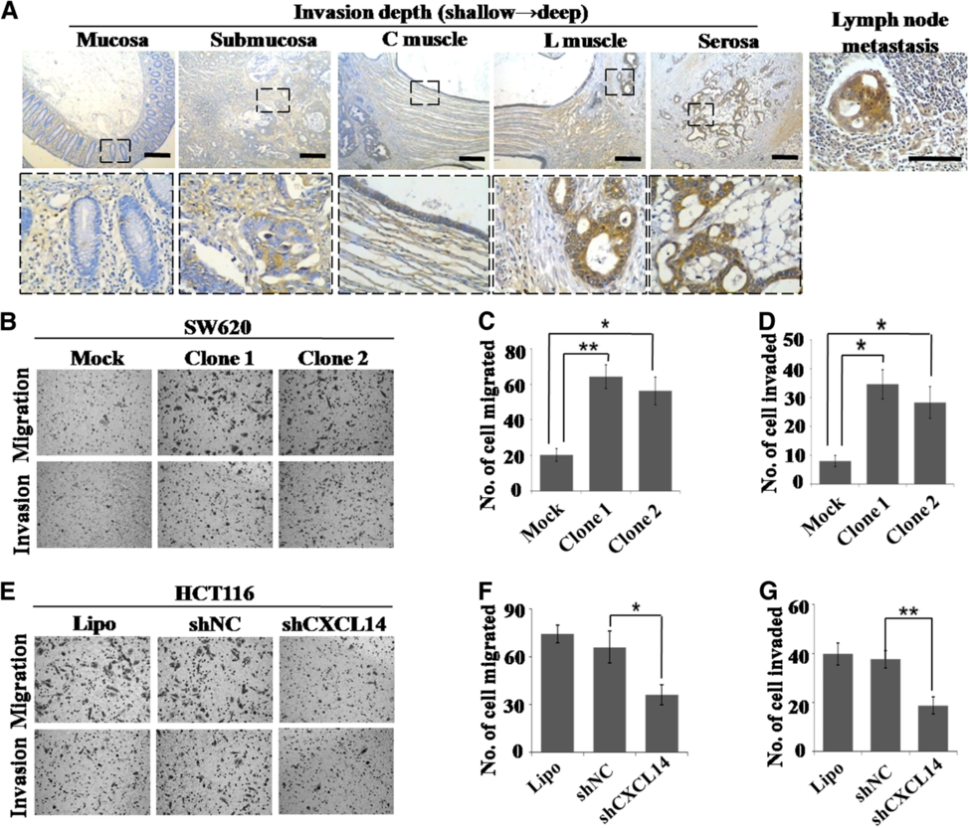
**CXCL14 promoted migration and invasion of SW620 cells. *A, ***the deeply invading carcinoma cells were more intensely stained with CXCL14 than those in mucosal region. ‘C muscle’ refers to circular muscle; ‘L muscle’ refers to longitudinal muscle. The cancer cells in the lymph node metastases were also CXCL14-immunopositive. ***B***-***D, ***a 2-chamber assay was used for evaluation of the migration/invasion ability of SW620 subclones. Over-expression of CXCL14 significantly increased the migration and invasion abilities. The number of migrated (*C*) and invaded (*D*) cells from five random fields were counted and presented with cell numbers. ***E***-***G, ***motility of HCT116 transfected with shCXCL14 or shNC were compared by transwell assay (*F*) and Matrigel assay (*G*), respectively. Column, mean; bars, SE (from triplicates). Student’s *t* test was used for the statistical analyses. *, P < 0.05; **, P < 0.01.

When analyzing the levels of CXCL14 expression in colorectal carcinoma tissues by immunohistochemistry, we found an interesting phenomenon that the number of CXCL14-immunopositive carcinoma cells increased as carcinoma cells invaded deeply and the tumor regions that were more close to the invasion front showed stronger CXCL14 immunoreactivity. Furthermore, the majority of the cancer cells in the lymph node metastases were also CXCL14-immunopositive (Figure 5A). These phenomena suggested CXCL14 was involved in CRC invasion and metastasis. Several *in vitro* studies were performed to obtain further validation. SW620 subclones (clone 1 and clone 2) migrated faster than control cells through uncoated membranes in the modified Boyden chamber assays, and were more invasive than control cells as demonstrated by Martrigel invasion. The numbers of migrated SW620 cells of CXCL14-transfeted groups were 64 ± 7 and 56 ± 8 for clone 1 and clone 2, respectively. Meanwhile the number of migrated cells in mock vector-transfected cells was 20 ± 4. Further, CXCL14 induced SW620 cell invasion by more than threefold when compared with the control groups: 35 ± 5 (clone 1), 28 ± 6 (clone 2) *versus* 8 ± 2 (mock control) (Figure 5B-D). In addition, treatment with rhCXCL14 protein also accelerated SW620 cell migration and invasion (Additional file 2: Figure S2D, E). However, shRNA-mediated inhibition of CXCL14 expression in HCT116 cells caused a significant decrease in cell migration and invasion (Figure 5E-G). These results demonstrated the functional potential of CXCL14 in promoting colorectal cancer cell migration and invasion.

## Discussion

CXCL14, a novel non-ELR chemokine with yet uncharacterised receptor, has been reported to be associated with tumor progression and metastasis [15,16,30]. In this study, we found that CXCL14 was frequently up-regulated in colorectal tumors compared with the adjacent normal colorectal mucosa. This finding is consistent with previous studies that CXCL14 expression was elevated in tumor tissues compared with adjacent normal tissues [19,20,30]. To the best of our knowledge, this is the first study that analyzes the expression of CXCL14 protein in colorectal carcinoma by immunohistochemistry using surgically resected neoplastic tissues. Furthermore, in early stage I colorectal cancer, CXCL14 expression was up-regulated compared with adjacent normal mucosa, suggesting that CXCL14 over-expression is an early event in the development of CRC. It is intriguing to speculate on possible reasons for these high expression levels of CXCL14 during tumorigenesis. Immunohistochemical analysis showed that CXCL14 expression was mainly located in malignant colorectal epithelial cells, and partially in stroma cells. Hence, it could be postulated that CXCL14, similar to other chemokines, is likely to signal through a G-coupled receptor and contributes to progression of CRC via both autocrine and paracrine pathways. Additionally, the levels of CXCL14 expression were significantly associated with some clinicopathologic factors including TNM stage, histodifferentiation, and tumor size. Previous studies have reported that these factors are possible predictors of early recurrence and cancer-death [31-33]. Among them, TNM stage is the strongest predictor of survival for patients with colorectal cancer [32]. The correlation between CXCL14 expression and these factors implies that elevated expression of CXCL14 may be associated with poor patient outcome.

Multivariate analysis showed that elevated CXCL14 level in patients with stage I/II colorectal carcinoma was an independent risk factor for developing recurrence, and that the level of CXCL14 expression in patients with stage III/IV colorectal carcinomas was inversely correlated with duration of survival. CXCL14 is therefore a useful prognostic factor for predicting the outcome of patients with CRC who have had a surgical resection of their tumor. Therefore, patients with colorectal carcinoma showing elevated CXCL14 expression should be carefully followed-up, while those with no or low CXCL14 expression should avoid being overtreated. In addition, tumors with high CXCL14 expression had a worse prognosis, even within the same clinicopathologic stage. The staging system, which is based on the extent of the tumor spread at the time of primary surgical treatment, does not always account for the aggressiveness of the tumor itself. From a clinical perspective, the ability to predict outcome within the same clinicopathologic stage is the key to individualizing treatment options. CXCL14, to some degree, helps makes up for the fact that TNM stage *per se* cannot predict the outcome of patients with the same clinical stage. Although clinicopathologic data dealing with CXCL14 expression at the protein level are currently scarce, increasing attention has been paid to the prognostic significance of CXCL14 in other diseases. Using antibody arrays, CXCL14 has been identified as a potential diagnostic marker of hepatocellular carcinoma [34]. Additionally, CXCL14 have been suggested to be a poor prognostic marker in papillary thyroid carcinoma by qPCR analysis [20].

The functional role(s) of CXCL14 in tumor biology has been addressed in a few previous reports [15,17]. In the present study, immunohistochemical analyses and a number of *in vitro* studies were performed to validate the aggressive role of CXCL14 in colorectal cancer progression. The observation that there was good correlation between CXCL14 levels and Ki67 in human colorectal cancer suggested that CXCL14 was involved in human colorectal cancer cell proliferation. Indeed, we found that CXCL14 enhanced malignant colorectal cell proliferation *in vitro*. Consistent with these findings, CAF-derived CXCL14 was reported to exert paracrine stimulatory effects on the proliferation of the prostate cancer cell line LNCaP [15]. Meanwhile, rhCXCL14 was shown to enhance the proliferation of MCF-7 breast cancer cell accompanied by a robust increase in ERK phosphorylation in an autocrine manner [35]. Intriguingly, CXCL14 expression was observed to be restricted to the myoepithelial cells in ductal carcinoma *in situ* and the tumor epithelial cells in invasive breast carcinoma, suggesting CXCL14 might be converted into an autocrine factor from a paracrine factor [30].

In almost all the specimens studied, the deeply invaded cancer cells were more intensely stained than those in mucosal regions. Additionally, the number of CXCL14-positive cancer cells increased as cancer cells invaded deeply, with more than 90% of the cells in the regions of the muscularis propria and subserosa immunopositive. Furthermore, almost all the cancer cells in the lymph node metastases were also CXCL14-immunopositive. These results suggest CXCL14 may be involved in the process of invasion and metastasis, especially lymphatic permeation and nodal metastasis of colorectal cancer cells. *In vitro* studies further validated the role of CXCL14 in colorectal cancer cell migration and invasion. The underlying mechanisms of CXCL14-enhanced colorectal cancer cell migration and invasion are not clear. Recent reports showed that activation of chemokine signaling could promote tumor cell metastasis by regulating polymerization of intracellular actins, inducing formation of pseudopodia, and facilitating extracellular matrix degradation and remodeling [36,37]. These findings are consistent with previous studies that implicated altered chemokine expression levels as an indicator of progression to tumorigenesis and metastasis capacity [38,39]. However, few studies have reported the potential of CXCL14 in colorectal carcinoma initiation and progression. In contrast to previous studies in which CXCL14 was reported to be deficient in tumors and acted as a tumor suppressor [17,18], CXCL14 is recently identified as a novel potential cancer-stimulatory protein [20]. Results obtained from the present study together with others indicate a need for further analysis of possible tumor type- and stage-specific effects of CXCL14 in other tumors [15,17,30]. The mechanisms through which CXCL14 promotes or inhibits tumor progression need to be further analyzed.

A series of topics worthy of further studies are suggested by the present findings. Firstly, identification of the receptor for CXCL14 is pressingly needed. The “seed and soil” theory that target organs releasing specific chemokines attract tumor cells bearing corresponding receptors has provided an acceptable explanation for many important roles of chemokine receptors in cancer metastasis [37,40]. Analyses of the effects of GPCR inhibitors with known target profiles and GPCR profiling of CXCL14-responsive and -nonresponsive cells, will possibly be useful for receptor identification. Identification of the factor(s) that cause up-regulation of CXCL14 in tumor epithelial cells is another relevant issue warranting further analyses. Understanding the mechanisms through which the chemokine CXCL14 promotes cell transformation and tumor progression is key to effective therapy. We examined the expression pattern of CXCL14 in CRC and its clinical value based on a small number of cases. Thus, there is a requirement for further investigations to clarify the correlation of CXCL14 expression with the clinical outcome of CRC with a larger patient cohort.

## Conclusion

In summary, these results provide the first definitive immunohistochemical evidence that CXCL14 expression is up-regulated in colorectal cancer. Additionally, we report the potential clinical value of understanding CXCL14 expression in patients with CRC. On the one hand, CXCL14 could be used as a prognostic factor for poor disease outcome. On the other hand, our studies suggest that CXCL14 expression may be beneficial for individualizing treatment options for CRC patients. One of the challenges to effective therapy lies in understanding the mechanisms through which the chemokine CXCL14 promotes cell transformation and tumor progression. Understanding these mechanisms is essential for developing new therapeutic strategies to effectively inhibit cancer growth and metastasis. Taken together, our study can form the basis for future potential novel tumor therapy.

## Abbreviations

CXCL: CXC chemokine ligand; CXCR: CXC chemokine receptor; CRC: Colorectal carcinoma; CAF: Cancer-associated fibroblast; DFS: Disease-free survival; EMT: Epithelial-mesenchymal transition; FFPE: Formalin-fixed paraffin-embedded; HNSCC: Head and neck squamous cell carcinomas; OS: Overall survival; PTC: Papillary thyroid carcinoma; TNM: Tumor-node-metastasis.

## Competing interests

The authors declare that they have no competing interests.

## Authors’ contributions

HC, ZJ conceived of the study, participated in the design of the study, carried out the immunohistochemistry assays, performed the statistical analysis and draft the manuscript. YX, DD, XK, LF, LQ and NE participated in the design of the study, collected the formalin-fixed and paraffin-embedded CRC patient tissues and related clinicpathologic information and performed the statistical analysis. LR, LY, CL and DL carried out the molecular genetic studies, participated in the sequence alignment, carried out the immunohistochemistry assays and drafted the manuscript. All authors read and approved the final manuscript.

## Supplementary Material

Additional file 1 **Figure S1.** The validation of the specificity of the antibody to CXCL14. *A*, Immunohistochemical staining for colorectal cancer specimens incubated with IgG or CXCL14-specific antibody. To validate the specificity, the antibody (ProteinTech) to CXCL14 was pre-incubated with recombinant human CXCL14 (PeproTech) for 1 h prior to applying to tissues. *B*, some samples were randomly selected and immunostaining experiments were done using both Proteintech’s and Abcam’s anti-CXCL14 antibodies in serial sections. Click here for file

Additional file 2 **Figure S2.** Effects of recombinant human CXCL14 on colorectal cancer cell proliferation and motility. *A*-*C*, SW620 cells were treated with rhCXCL14 at indicated doses. Proliferation of SW620 cells were measured by BrdU assay (*A*), MTT assay (*B*), and colony formation assay (*C*). *D*-*E*, rhCXCL14 significantly increased the cells’ migration and invasion abilities. The number of migrated (*D*) and invaded (*E*) cells from five random fields were counted and presented with cell numbers. Column, mean; bars, SE (from triplicates). Student’s *t* test was used for the statistical analyses. *, P < 0.05; **, P < 0.01. Click here for file
