# Supplementary figures and images for: Chemokine CXCL14 is associated with prognosis in patients with colorectal carcinoma after curative resection
Source: J Transl Med. 2013 Jan 7;11:6. doi: 10.1186/1479-5876-11-6 (PMC3551837; doi:10.1186/1479-5876-11-6)

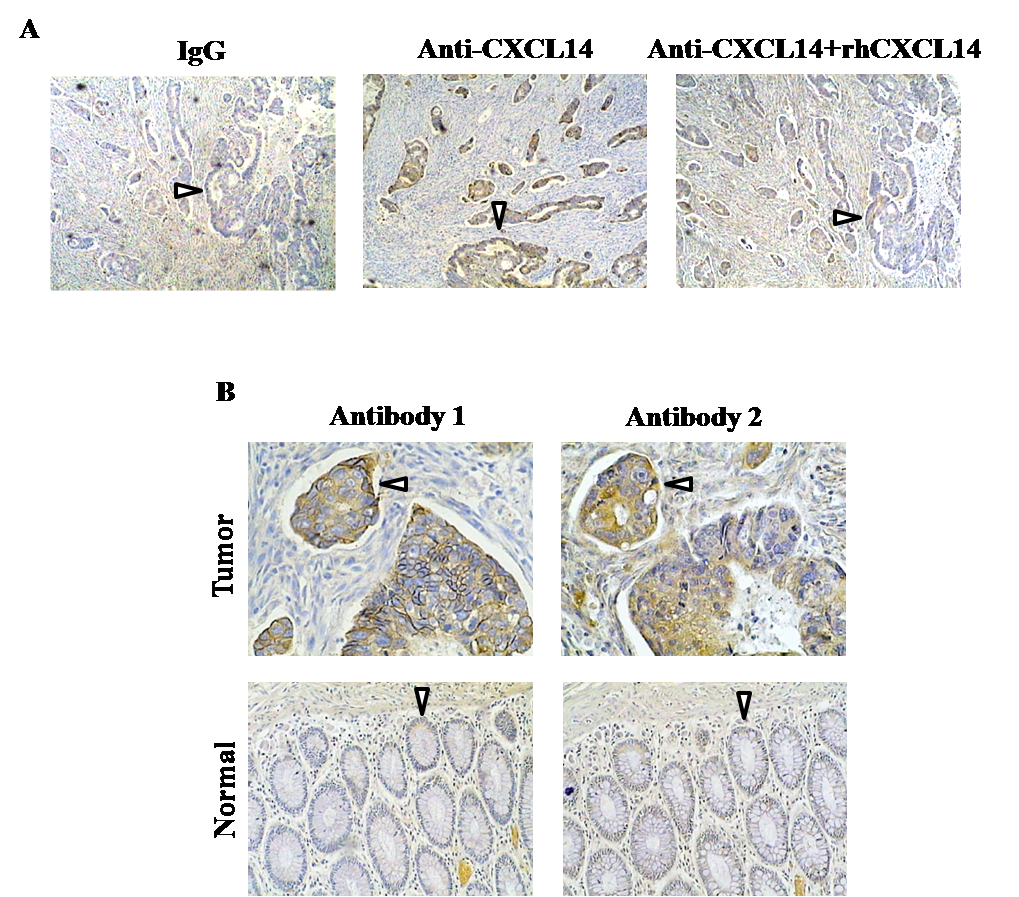

Supplement: Additional file 1 — Figure S1. The validation of the specificity of the antibody to CXCL14. A, Immunohistochemical staining for colorectal cancer specimens incubated with IgG or CXCL14-specific antibody. To validate the specificity, the antibody (ProteinTech) to CXCL14 was pre-incubated with recombinant human CXCL14 (PeproTech) for 1 h prior to applying to tissues. B, some samples were randomly selected and immunostaining experiments were done using both Proteintech’s and Abcam’s anti-CXCL14 antibodies in serial sections. [file 1479-5876-11-6-S1.tiff]

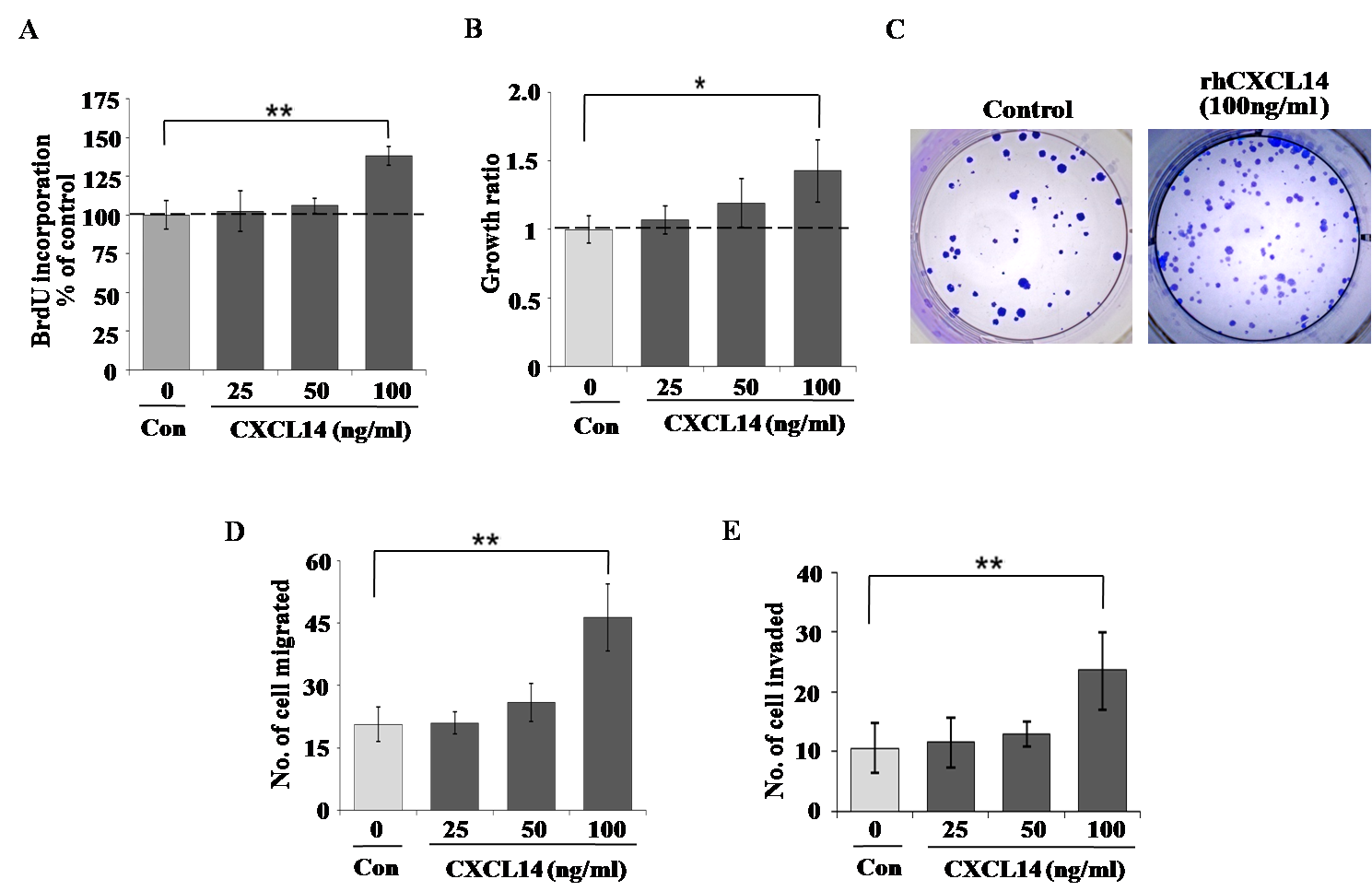

Supplement: Additional file 2 — Figure S2. Effects of recombinant human CXCL14 on colorectal cancer cell proliferation and motility. A-C, SW620 cells were treated with rhCXCL14 at indicated doses. Proliferation of SW620 cells were measured by BrdU assay (A), MTT assay (B), and colony formation assay (C). D-E, rhCXCL14 significantly increased the cells’ migration and invasion abilities. The number of migrated (D) and invaded (E) cells from five random fields were counted and presented with cell numbers. Column, mean; bars, SE (from triplicates). Student’s t test was used for the statistical analyses. *, P < 0.05; **, P < 0.01. [file 1479-5876-11-6-S2.tiff]
